# Supplementary material for: Trauma-informed care (TIC) in low- and middle-income countries: A scoping review of organisational implementation efforts
Source: Glob Ment Health (Camb). 2025 Dec 10;12:e148. doi: 10.1017/gmh.2025.10111 (PMC12720385; doi:10.1017/gmh.2025.10111)
Supplement: Maiorano et al. supplementary material [file S2054425125101118sup001.zip › Supplementary_File_1._List_of_included_countries.docx]

S1 Appendix. Countries for inclusion

| Afghanistan | Congo, Rep. | Iraq | Morocco | South Sudan |
| --- | --- | --- | --- | --- |
| Albania | Costa Rica | Jamaica | Mozambique | Sri Lanka |
| Algeria | Côte d'Ivoire | Jordan | Myanmar | St. Kitts and Nevis |
| American Samoa | Croatia | Kazakhstan | Namibia | St. Lucia |
| Angola | Cuba | Kenya | Nauru | St. Vincent and the Grenadines |
| Argentina | Djibouti | Kiribati | Nepal | Sudan |
| Armenia | Dominica | Korea, Dem. Rep. | Nicaragua | Suriname |
| Azerbaijan | Dominican Republic | Kosovo | Niger | Syrian Arab Republic |
| Bangladesh | Ecuador | Kyrgyz Republic | Nigeria | Tajikistan |
| Belarus | Egypt, Arab Rep. | Lao PDR | North Macedonia | Tanzania |
| Belize | El Salvador | Latvia | Pakistan | Thailand |
| Benin | Equatorial Guinea | Lebanon | Palau | Timor-Leste |
| Bhutan | Eritrea | Lesotho | Panama | Togo |
| Bolivia | Eswatini | Liberia | Papua New Guinea | Tonga |
| Bosnia and Herzegovina | Ethiopia | Libya | Paraguay | Tunisia |
| Botswana | Fiji | Lithuania | Peru | Turkey |
| Brazil | Gabon | Madagascar | Philippines | Turkmenistan |
| Bulgaria | Gambia, The | Malawi | Romania | Tuvalu |
| Burkina Faso | Georgia | Malaysia | Russian Federation | Uganda |
| Burundi | Ghana | Maldives | Rwanda | Ukraine |
| Cabo Verde | Grenada | Mali | Samoa | Uruguay |
| Cambodia | Guatemala | Marshall Islands | São Tomé and Principe | Uzbekistan |
| Cameroon | Guinea | Mauritania | Senegal | Vanuatu |
| Central African Republic | Guinea-Bissau | Mauritius | Serbia | Venezuela, RB |
| Chad | Guyana | Mayotte | Serbia and Montenegro (former) | Vietnam |
| Chile | Haiti | Mexico | Seychelles | West Bank and Gaza |
| China | Honduras | Micronesia, Fed. Sts. | Sierra Leone | Yemen, Rep. |
| Colombia | India | Moldova | Solomon Islands | Zambia |
| Comoros | Indonesia | Mongolia | Somalia | Zimbabwe |
| Congo, Dem. Rep. | Iran, Islamic Rep. | Montenegro | South Africa |  |
