# Supplementary material for: Trauma-informed care (TIC) in low- and middle-income countries: A scoping review of organisational implementation efforts
Source: Glob Ment Health (Camb). 2025 Dec 10;12:e148. doi: 10.1017/gmh.2025.10111 (PMC12720385; doi:10.1017/gmh.2025.10111)
Supplement: Maiorano et al. supplementary material [file S2054425125101118sup001.zip › Supplementary_File_2._Sample_search_string.docx]

S2 Appendix. Sample search string

Web of Science 1,912 results

(((organizational OR organisational) NEAR/2 (change* OR innovation*)) OR "quality improvement*")

AND

("trauma informed" OR “trauma awar*” OR “trauma sensitive*” OR Safe* OR Trust* OR Transparency OR (peer NEAR/2 support*) OR Collaboration* OR Mutuality OR Empower* OR Voice* OR Choice* OR Resilience* OR Stereotype* OR Bias* OR “discrimination”)

AND

(africa OR "latin america" OR caribbean OR "west indies" OR "eastern europe" OR soviet OR "south america" OR "middle east" OR (low* NEAR/3 middle NEAR/3 countr*) OR "LMIC" OR "LMICs" OR "LAMI" OR ((transitional OR develop*) NEAR/3 (countr* OR nation* OR world)) OR afghanistan OR albania OR algeria OR samoa OR angola OR argentina OR armenia OR azerbaijan OR bangladesh OR belarus OR belize OR benin OR bhutan OR bolivia OR bosnia OR herzegovina OR botswana OR brazil OR bulgaria OR "burkina faso" OR burundi OR "cabo verde" OR cambodia OR cameroon OR "central african republic" OR chad OR chile OR china OR colombia OR comoros OR congo OR "costa rica" OR (cote NEAR/2 ivoire) OR "ivory coast" OR cuba OR djibouti OR dominica* OR ecuador OR egypt OR salvador OR eritrea OR ethiopia OR fiji OR gabon OR gambia OR georgia OR ghana OR grenada OR guatemala OR guinea OR guyana OR haiti OR honduras OR india OR indonesia OR iran OR iraq OR jamaica OR jordan OR kazakhstan OR kenya OR kiribati OR korea OR kosovo OR kyrgyz OR laos OR latvia OR lebanon OR lesotho OR liberia OR libya OR lithuania OR macedonia OR madagascar OR malawi OR malaysia OR maldives OR mali OR "marshall islands" OR mauritania OR mauritius OR mexico OR micronesia OR moldova OR mongolia OR montenegro OR morocco OR mozambique OR myanmar OR namibia OR nepal OR nicaragua OR niger OR nigeria OR pakistan OR palau OR panama OR papua new guinea OR paraguay OR peru OR philippines OR romania OR russia OR rwanda OR "sao tome" OR senegal OR serbia OR seychelles OR "sierra leone" OR "solomon islands" OR somalia OR somaliland OR "sri lanka" OR "st kitts" OR "st lucia" OR "st vincent" OR grenadines OR sudan OR suriname OR swaziland OR syria OR tajikistan OR tanzania OR thailand OR "timor leste" OR togo OR tonga OR tunisia OR turkey OR turkmenistan OR tuvalu OR uganda OR ukraine OR uruguay OR uzbekistan OR vanuatu OR venezuela OR vietnam OR "west bank" OR gaza OR yemen OR zambia OR zimbabwe)
