# Supplementary material for: Trauma-informed care (TIC) in low- and middle-income countries: A scoping review of organisational implementation efforts
Source: Glob Ment Health (Camb). 2025 Dec 10;12:e148. doi: 10.1017/gmh.2025.10111 (PMC12720385; doi:10.1017/gmh.2025.10111)
Supplement: Maiorano et al. supplementary material [file S2054425125101118sup001.zip › Supplementary_File_3._SAMHSA_principles_defined.docx]

S3 Appendix. SAMHSA principles defined

| **Principle** | **SAMSHA Definition** | **Inclusion/Exclusion Examples** |
| --- | --- | --- |
| Safety | “Throughout the organization, staff and the people they serve, whether children or adults, feel physically and psychologically safe; the physical setting is safe and interpersonal interactions promote a sense of safety. Understanding safety as defined by those served is a high priority.” | *Inclusion:* Practices to improve workplace for staff, increase client comfort/safe access to services, support client and staff interactions    *Exclusion:* Improvements to medical procedures (e.g., surgical checklist, safe medicine/anaesthesia administration processes), implementation to decrease negative medical experiences (e.g., infection rates, staff errors) |
| Trustworthiness and transparency | “Organizational operations and decisions are conducted with transparency with the goal of building and maintaining trust with clients and family members, among staff, and others involved in the organization.” | *Inclusion:* Interventions to increase organisational trust or to make organisational decisions/governance more transparent |
| Peer support | “Peer support and mutual self-help are key vehicles for establishing safety and hope, building trust, enhancing collaboration, and utilizing their stories and lived experience to promote recovery and healing. The term “Peers” refers to individuals with lived experience of trauma, or in the case of children this may be family members of children who have experienced traumatic events and are key caregivers in their recovery. Peers have also been referred to as ‘trauma survivors’. | *Inclusion:* Organisational practices for peer supervision/support for staff, structures to allow clients to collaborate with each other |
| Collaboration and mutuality | Importance is placed on partnering and the leveling of power differences between staff and clients and among organizational staff from clerical and housekeeping personnel, to professional staff to administrators, demonstrating that healing happens in relationships and in the meaningful sharing of power and decision-making. The organization recognizes that everyone has a role to play in a trauma-informed approach. As one expert stated: ‘one does not have to be a therapist to be therapeutic.’” | *Inclusion:* Interventions/efforts to increase collaboration (e.g., increase collaboration between nurse and doctor staff) or to reduce power dynamics (e.g., structures for Client involvement in treatment choice) within organisations    *Exclusion:* QI efforts that use collaboration to achieve a non-TIC outcome (e.g., collaborative registry for kidney disease, collaboration to reduce infection) unless the intervention in the paper focuses on supporting the collaboration process (e.g., a QI intervention to increase collaboration to reduce infection) |
| Empowerment, voice and choice | “Throughout the organization and among the clients served, individuals’ strengths and experiences are recognized and built upon. The organization fosters a belief in the primacy of the people served in resilience, and in the ability of individuals, organizations, and communities to heal and promote recover from trauma. The organization understand that the experience of trauma may be a unifying aspect in the lives of those who run the organization, who provider the services, and/or who come to the organization for assistance and support. As such, operations, workforces development and services are organized to foster empowerment for staff and clients alike. Organizations understand the importance of power differentials and ways in which clients, historically, have been diminished in voice and choice and are often recipients of coercive treatment. Clients are supported in shared decision-making, choice, and goal setting to determine the plan of action they need to heal and move forward. They are supported in cultivating self-advocacy skills. Staff are facilitators of recovery rather than controllers of recovery. Staff are empowered to do their work as well as possible by adequate organizational support. This is a parallel process as staff need to feel safe, as much as people receiving services.” | *Inclusion:* Increasing communities/Clients voice in organisation (e.g., use of participatory methods), increasing staff voice in decision making (e.g., changes to decision making process)    *Exclusion:* QI efforts that use empowerment, voice, and choice to achieve a non-TIC outcome (e.g., patients’ advice on best method for diabetes treatment) unless the intervention in the paper focuses on supporting the use of empowerment, voice and choice method (e.g., a QI intervention to increase patient voice in diabetes treatment) |
| Cultural, historical, and gender issues | “The organization actively moves past cultural stereotypes and biases (e.g., based on race, ethnicity, sexual orientation, age, religion, gender-identity, geography, etc.); offers, access to gender responsive services; leverages the healing value of traditional cultural connections; incorporates policies, protocols, and processes that are responsive to the racial, ethnic, and cultural needs of individuals served; and recognizes and addresses historic trauma.” | *Inclusion:* Organisational changes to reduce the influence of bias/discrimination in services based on protected categories (those listed by SAMSHA; e.g., maternal health/reduction of maternal mortality, HIV/AIDS reduction/support, reduction of discrimination/violence)    *Exclusion:* Organisational changes that do not relate to a historically oppressed identity (e.g., neonatal health). |
