# Supplementary material for: Trauma-informed care (TIC) in low- and middle-income countries: A scoping review of organisational implementation efforts
Source: Glob Ment Health (Camb). 2025 Dec 10;12:e148. doi: 10.1017/gmh.2025.10111 (PMC12720385; doi:10.1017/gmh.2025.10111)
Supplement: Maiorano et al. supplementary material [file S2054425125101118sup001.zip › Supplementary_File_4._References_of_included_articles.docx]

**References List: Included Articles**

1. Abedi G, Haghgoshayie E, Hasanpoor E, Etemadi J, Nazari M, Vejdani R. Improvement of violence management among nurses in Iran: The best practice implementation project in a health promoting hospital. PloS one. 2023;18(11):e0284758.

2. Abuya T, Ndwiga C, Ritter J, Kanya L, Bellows B, Binkin N, et al. The effect of a multi-component intervention on disrespect and abuse during childbirth in Kenya. BMC pregnancy and childbirth. 2015;15:1–14.

3. Abuya T, Njuki R, Warren CE, Okal J, Obare F, Kanya L, et al. A policy analysis of the implementation of a reproductive health vouchers program in Kenya. BMC Public Health. 2012;12:1–14.

4. Adam M, Makobu N, Kamiru W, Mbugua S, Mailu F. Coping with COVID: Developing a Rapid-cycle Frontline Quality-improvement Process to Support Employee Well-being and Drive Institutional Responsiveness in a Tertiary Care Faith-based Hospital in Rural Kenya. AMERICAN JOURNAL OF TROPICAL MEDICINE AND HYGIENE. 2021;105(2):372–4.

5. Agrawal S, Puri M, Singh A, Sehrawat S, Sood S, Choudhary K, et al. Increasing postpartum IUCD coverage through a QI initiative: a step towards reducing the unmet need of postpartum contraception. BMJ open quality [Internet]. 2021;10(Suppl 1). Available from: <https://elib.tcd.ie/login?url=https://search.ebscohost.com/login.aspx?direct=true&db=cmedm&AN=34344746&site=ehost-live>

6. AHI PHIT Partnership Collaborative, Manzi A, Hirschhorn L, Sherr K, Chirwa C, Baynes C, et al. Mentorship and coaching to support strengthening healthcare systems: lessons learned across the five Population Health Implementation and Training partnership projects in sub-Saharan Africa. BMC HEALTH SERVICES RESEARCH. 2017;17.

7. Ahmed S, Khan MM. A maternal health voucher scheme: what have we learned from the demand-side financing scheme in Bangladesh? Health policy and planning. 2011;26(1):25–32.

8. Ahmed SM. Capability development among the ultra-poor in Bangladesh: a case study. Journal of health, population, and nutrition. 2009;27(4):528–35.

9. Algunmeeyn A, Alrawashdeh M, Alhabashneh H. Benefits of applying for hospital accreditation: The perspective of staff. Journal of nursing management. 2020;28(6):1233–40.

10. Alhassan R, Nketiah-Amponsah E, Arhinful D. Design and implementation of community engagement interventions towards healthcare quality improvement in Ghana: a methodological approach. HEALTH ECONOMICS REVIEW. 2016;6.

11. Alhassan RK, Nketiah-Amponsah E, Spieker N, Arhinful DK, Rinke de Wit TF. Assessing the impact of community engagement interventions on health worker motivation and experiences with clients in primary health facilities in Ghana: a randomized cluster trial. PloS one. 2016;11(7):e0158541.

12. Alhassan RK, Nketiah-Amponsah E, Spieker N, Arhinful DK, Rinke de Wit TF. Perspectives of frontline health workers on Ghana’s National Health Insurance Scheme before and after community engagement interventions. BMC health services research. 2016;16:1–11.

13. Alwano MG, Bachanas P, Block L, Roland M, Sento B, Behel S, et al. Increasing knowledge of HIV status in a country with high HIV testing coverage: Results from the Botswana Combination Prevention Project. PloS one. 2019;14(11):e0225076.

14. An S, George A, LeFevre A, Mpembeni R, Mosha I, Mohan D, et al. Program synergies and social relations: implications of integrating HIV testing and counselling into maternal health care on care seeking. BMC PUBLIC HEALTH. 2015;15.

15. Anaba U, Sam-Agudu N, Ramadhani H, Torbunde N, Abimiku A, Dakum P, et al. Missed opportunities for early infant diagnosis of HIV in rural North-Central Nigeria: A cascade analysis from the INSPIRE MoMent study. PLOS ONE. 2019;14(7).

16. Arem H, Nakyanjo N, Kagaayi J, Mulamba J, Nakigozi G, Serwadda D, et al. Peer health workers and AIDS care in Rakai, Uganda: a mixed methods operations research evaluation of a cluster-randomized trial. AIDS Patient Care and STDs. 2011;25(12):719–24.

17. Awoonor-Williams JK, Feinglass ES, Tobey R, Vaughan-Smith MN, Nyonator FK, Jones TC. Bridging the Gap Between Evidence-based Innovation and National Health-sector Reform in Ghana. Studies in Family Planning. 2004;35(3):161–77.

18. Awoonor-Williams JK, Sory EK, Nyonator FK, Phillips JF, Wang C, Schmitt ML. Lessons learned from scaling up a community-based health program in the Upper East Region of northern Ghana. Global Health: Science and Practice. 2013;1(1):117–33.

19. Baatiema L, Skovdal M, Rifkin S, Campbell C. Assessing participation in a community-based health planning and services programme in Ghana. BMC health services research. 2013;13:1–13.

20. Bacha JM, Aririguzo LC, Mng’ong’o V, Malingoti B, Wanless RS, Ngo K, et al. The Standardized Pediatric Expedited Encounters for ART Drugs Initiative (SPEEDI): description and evaluation of an innovative pediatric, adolescent, and young adult antiretroviral service delivery model in Tanzania. BMC infectious diseases. 2018;18(1):448.

21. Bailey P, Binh H, Bang H. Promoting accountability in obstetric care: use of criteria-based audit in Viet Nam. Global public health. 2010;5(1):62–74.

22. Baker U, Petro A, Marchant T, Peterson S, Manzi F, Bergström A, et al. Health workers’ experiences of collaborative quality improvement for maternal and newborn care in rural Tanzanian health facilities: A process evaluation using the integrated’Promoting Action on Research Implementation in Health Services’ framework. PLoS One. 2018;13(12):e0209092.

23. Baral G. An assessment of the safe delivery incentive program at a tertiary level hospital in Nepal. Journal of Nepal Health Research Council. 2012;

24. Basinga P, Gertler PJ, Binagwaho A, Soucat AL, Sturdy J, Vermeersch CM. Effect on maternal and child health services in Rwanda of payment to primary health-care providers for performance: an impact evaluation. The Lancet. 2011;377(9775):1421–8.

25. Bazant E, Sarkar S, Banda J, Kanjipite W, Reinhardt S, Shasulwe H, et al. Effects of a performance and quality improvement intervention on the work environment in HIV-related care: a quasi-experimental evaluation in Zambia. HUMAN RESOURCES FOR HEALTH. 2014;12.

26. Bedelu M, Ford N, Hilderbrand K, Reuter H. Implementing antiretroviral therapy in rural communities: the Lusikisiki model of decentralized HIV/AIDS care. The Journal of infectious diseases. 2007;196(Supplement_3):S464–8.

27. Behdjat H, Rifkin SB, Tarin E, Sheikh MR. A new role for Women Health Volunteers in urban Islamic Republic of Iran. Eastern Mediterranean health journal = La revue de sante de la Mediterranee orientale = al-Majallah al-sihhiyah li-sharq al-mutawassit. 2009;15(5):1164–73.

28. Beima-Sofie K, Wagner AD, Soi C, Liu W, Tollefson D, Njuguna IN, et al. Providing “a beam of light to see the gaps”: determinants of implementation of the Systems Analysis and Improvement Approach applied to the pediatric and adolescent HIV cascade in Kenya. Implementation science communications. 2022;3(1):73.

29. Bellows B, Mackay A, Dingle A, Tuyiragize R, Nnyombi W, Dasgupta A. Increasing Contraceptive Access for Hard-to-Reach Populations With Vouchers and Social Franchising in Uganda. GLOBAL HEALTH-SCIENCE AND PRACTICE. 2017;5(3):446–55.

30. BetterBirth Trial Grp, Semrau K, Hirschhorn L, Delaney M, Singh V, Saurastri R, et al. Outcomes of a Coaching-Based WHO Safe Childbirth Checklist Program in India. NEW ENGLAND JOURNAL OF MEDICINE. 2017;377(24):2313–24.

31. Bhardwaj S, Barron P, Pillay Y, Treger-Slavin L, Robinson P, Goga A, et al. Elimination of mother-to-child transmission of HIV in South Africa: Rapid scale-up using quality improvement. SAMJ SOUTH AFRICAN MEDICAL JOURNAL. 2014;104(3):239–43.

32. Bhuiya A, Hanifi S, Hoque S. Unlocking community capability through promotion of self-help for health: experience from Chakaria, Bangladesh. BMC HEALTH SERVICES RESEARCH. 2016;16.

33. Bisnauth M, Davies N, Monareng S, Struthers H, McIntyre J, Rees K. Exploring healthcare workers’ experiences of managing patients returning to HIV care in Johannesburg, South Africa. GLOBAL HEALTH ACTION. 2022;15(1).

34. Bitewulign B, Abdissa D, Mulissa Z, Kiflie A, Abate M, Biadgo A, et al. Using the WHO safe childbirth checklist to improve essential care delivery as part of the district-wide maternal and newborn health quality improvement initiative, a time series study. BMC HEALTH SERVICES RESEARCH. 2021;21(1).

35. Björkman M, Svensson J. Power to the people: evidence from a randomized field experiment on community-based monitoring in Uganda. The Quarterly Journal of Economics. 2009;124(2):735–69.

36. Bogren M, Mwambali S, Berg M. Contextual factors influencing a training intervention aimed at improved maternal and newborn healthcare in a health zone of the Democratic Republic of Congo. PLOS ONE. 2021;16(11).

37. Bond L, Farrar J, Borg RC, Keegan K, Journeay K, Hansen N, et al. Alternate delivery platforms and implementation models for bringing evidence-based behavioral interventions to scale for youth facing adversity: a case study in West Africa. Implementation science communications. 2022;3(1):16.

38. Borem P, de Cássia Sanchez R, Torres J, Delgado P, Petenate AJ, Peres D, et al. A Quality Improvement Initiative to Increase the Frequency of Vaginal Delivery in Brazilian Hospitals. Obstetrics and gynecology. 2020;135(2):415–25.

39. Boustani N, Sayegh M, Boustany Z. Strengthening Public Institutions and Social Inclusion of Vulnerable Groups in A Developing Country - Innovation in Organizations and Artificial intelligence Implications. PACIFIC ASIA JOURNAL OF THE ASSOCIATION FOR INFORMATION SYSTEMS. 2022;14(3):77–102.

40. Boyd A, Ogbanufe O, Onyenuobi C, Mgbakor I, Bachanas P, Olupitan O, et al. Scale-up of antiretroviral treatment access among people living with HIV in Rivers State, Nigeria, 2019--2020. AIDS. 2021;35(7):1127–34.

41. Boyd MA, Fwoloshi S, Minchella PA, Simpungwe J, Siansalama T, Barradas DT, et al. A national HIV clinical mentorship program: Enabling Zambia to accelerate control of the HIV epidemic. PLOS global public health. 2022;2(2):e0000074.

42. Boydell V, Nulu N, Hardee K, Gay J. Implementing social accountability for contraceptive services: lessons from Uganda. BMC Women’s Health. 2020;20:1–12.

43. Bradley J, Igras S. Improving the quality of child health services: participatory action by providers. INTERNATIONAL JOURNAL FOR QUALITY IN HEALTH CARE. 2005;17(5):391–9.

44. Bradley J, Jayanna K, Shaw S, Cunningham T, Fischer E, Mony P, et al. Improving the knowledge of labour and delivery nurses in India: a randomized controlled trial of mentoring and case sheets in primary care centres. BMC health services research. 2017;17:1–8.

45. Cadée F, Perdok H, Sam B, de Geus M, Kweekel L. “Twin2twin” an innovative method of empowering midwives to strengthen their professional midwifery organisations. Midwifery. 2013;29(10):1145–50.

46. Carvalho S, Asgedom A, Rose P. Whose voice counts? Examining government-donor negotiations in the design of Ethiopia’s large-scale education reforms for equitable learning. DEVELOPMENT POLICY REVIEW. 2022;40(5).

47. Cavallin F, Maziku D, Mkolomi R, Azzimonti G, Manenti F, Putoto G, et al. Changes in maternal and neonatal care after a quality improvement intervention in a sub-Saharan setting. The Journal of Maternal-Fetal & Neonatal Medicine. 2020;33(24):4076–82.

48. Chan AK, Mateyu G, Jahn A, Schouten E, Arora P, Mlotha W, et al. Outcome assessment of decentralization of antiretroviral therapy provision in a rural district of Malawi using an integrated primary care model. Tropical Medicine & International Health. 2010;15:90–7.

49. Chang LW, Kagaayi J, Nakigozi G, Ssempijja V, Packer AH, Serwadda D, et al. Effect of peer health workers on AIDS care in Rakai, Uganda: a cluster-randomized trial. PloS one. 2010;5(6):e10923.

50. Chaote P, Mwakatundu N, Dominico S, Mputa A, Mbanza A, Metta M, et al. Birth companionship in a government health system: a pilot study in Kigoma, Tanzania. BMC pregnancy and childbirth. 2021;21:1–17.

51. Chary A, Flood D, Austad K, Colom M, Hawkins J, Cnop K, et al. Accompanying indigenous Maya patients with complex medical needs: A patient navigation system in rural Guatemala. Healthcare (Amsterdam, Netherlands). 2018;6(2):144–9.

52. Chaturvedi S, De Costa A, Raven J. Does the Janani Suraksha Yojana cash transfer programme to promote facility births in India ensure skilled birth attendance? A qualitative study of intrapartum care in Madhya Pradesh. Global health action. 2015;8(1):27427.

53. Chopra M, Arora N, Sinha S, Holschneider S, Livesley N. Improving postpartum care in a large hospital in New Delhi, India. BMJ open quality. 2018;7(3):e000423.

54. Cofie L, Barrington C, Akaligaung A, Reid A, Fried B, Singh K, et al. Integrating community outreach into a quality improvement project to promote maternal and child health in Ghana. GLOBAL PUBLIC HEALTH. 2014;9(10):1184–97.

55. Colbourn T, Nambiar B, Bondo A, Makwenda C, Tsetekani E, Makonda-Ridley A, et al. Effects of quality improvement in health facilities and community mobilization through women’s groups on maternal, neonatal and perinatal mortality in three districts of Malawi: MaiKhanda, a cluster randomized controlled effectiveness trial. International health. 2013;5(3):180–95.

56. Colom M, Austad K, Sacuj N, Larson K, Rohloff P. Expanding access to primary healthcare for women through a microfinance institution: A case study from rural Guatemala. HEALTHCARE-THE JOURNAL OF DELIVERY SCIENCE AND INNOVATION. 2018;6(4):223–30.

57. Das A, Nawal D, Singh MK, Karthick M, Pahwa P, Shah MB, et al. Evaluation of the mobile nurse training (MNT) intervention–a step towards improvement in intrapartum practices in Bihar, India. BMC pregnancy and childbirth. 2017;17:1–11.

58. Das S, Abdulwahid E, Moisan A, De Jesus A. Use of quality improvement methodology to improve care of women with hypertensive disease in pregnancy and haemorrhage in Yemen (low-income, high-insecurity setting). BMJ OPEN QUALITY. 2022;11(4).

59. Dayekh A, Naseridine M, Dakroub F, Olleik A. Impact, obstacles and boundaries of patient partnership: A qualitative interventional study in Lebanon. PLOS ONE. 2022;17(7).

60.Debpuur C, Phillips JF, Jackson EF, Nazzar A, Ngom P, Binka FN. The impact of the Navrongo Project on contraceptive knowledge and use, reproductive preferences, and fertility. Studies in family planning. 2002;33(2):141–64.

61. Delaney MM, Maji P, Kalita T, Kara N, Rana D, Kumar K, et al. Improving adherence to essential birth practices using the WHO safe childbirth checklist with peer coaching: experience from 60 public health facilities in Uttar Pradesh, India. Global Health: Science and Practice. 2017;5(2):217–31.

62. Dhlamini L, Knight L, van Rooyen H, van Heerden A, Rotheram‐Borus MJ. Qualitative interviews with mentor mothers living with HIV: potential impacts of role and coping strategies. Journal of the International AIDS Society. 2012;15:17391.

63. Diaz-Rios C, Urbano-Canal N. Publicly Subsidized Private Schools in Developing Countries: Lessons from Colombia. EDUCATION POLICY ANALYSIS ARCHIVES. 2021;29.

64. Dickson KE, Ashton J, Smith JM. Does setting adolescent-friendly standards improve the quality of care in clinics? Evidence from South Africa. International Journal for Quality in Health Care. 2007;19(2):80–9.

65. Diniz CSG, d’Orsi E, Domingues RMSM, Torres JA, Dias MAB, Schneck CA, et al. Implementation of the presence of companions during hospital admission for childbirth: data from the Birth in Brazil national survey. Cadernos de saude publica. 2014;30:S140–53.

66. Doherty T, Chopra M, Nsibande D, Mngoma D. Improving the coverage of the PMTCT programme through a participatory quality improvement intervention in South Africa. BMC public health. 2009;9:1–9.

67. Domingues RMSM, Dias MAB, do Carmo Leal M. Women’s preference for a vaginal birth in Brazilian private hospitals: effects of a quality improvement project. Reproductive Health. 2022;20(Suppl 2):188.

68. Dougherty G, Boccanera R, Boyd M, Gantt T, Kasonka S, Kasonde P, et al. A Quality Improvement Collaborative for Adolescents Living With HIV to Improve Immediate Antiretroviral Therapy Initiation at 25 Health Facilities in Lusaka, Zambia. JANAC-JOURNAL OF THE ASSOCIATION OF NURSES IN AIDS CARE. 2021;32(6):701–12.

69. Dougherty G, Panya M, Madevu-Matson C, Anyalechi GE, Clarke K, Fayorsey R, et al. Reaching the first 90: improving inpatient pediatric provider-initiated HIV testing and counseling using a quality improvement collaborative strategy in Tanzania. Journal of the Association of Nurses in AIDS Care. 2019;30(6):682–90.

70. Dumont A, Gaye A, de Bernis L, Chaillet N, Landry A, Delage J, et al. Facility-based maternal death reviews: effects on maternal mortality in a district hospital in Senegal. BULLETIN OF THE WORLD HEALTH ORGANIZATION. 2006;84(3):218–24.

71. Dumont A, Fournier P, Abrahamowicz M, Traoré M, Haddad S, Fraser WD. Quality of care, risk management, and technology in obstetrics to reduce hospital-based maternal mortality in Senegal and Mali (QUARITE): a cluster-randomised trial. The Lancet. 2013;382(9887):146–57.

72. Dumont A, Gaye A, Mahé P, Bouvier‐Colle M. Emergency obstetric care in developing countries: impact of guidelines implementation in a community hospital in Senegal. BJOG: An International Journal of Obstetrics & Gynaecology. 2005;112(9):1264–9.

73. Duvall S, Thurston S, Weinberger M, Nuccio O, Fuchs-Montgomery N. Scaling up delivery of contraceptive implants in sub-Saharan Africa: operational experiences of Marie Stopes International. Global Health: Science and Practice. 2014;2(1):72–92.

74. Edward A, Osei-Bonsu K, Branchini C, Yarghal TS, Arwal SH, Naeem AJ. Enhancing governance and health system accountability for people centered healthcare: an exploratory study of community scorecards in Afghanistan. BMC health services research. 2015;15:1–15.

75. Emond A, Pollock J, Costa N da, Maranhão T, Macedo A. The effectiveness of community-based interventions to improve maternal and infant health in the Northeast of Brazil. Revista Panamericana de Salud Pública. 2002;12(2):101–10.

76. ERANet-LAC CODE Project Grp, Goldraij G, Tripodoro V, Aloisio M, Castro S, Gerlach C, et al. One chance to get it right: improving clinical handovers for better symptom control at the end of life. BMJ OPEN QUALITY. 2021;10(3).

77. Ersdal H, Mdoe P, Mduma E, Moshiro R, Guga G, Kvaloy J, et al. “Safer Births Bundle of Care” Implementation and Perinatal Impact at 30 Hospitals in Tanzania-Halfway Evaluation. CHILDREN-BASEL. 2023;10(2).

78. Eustache E, Gerbasi ME, Severe J, Fils-Aimé JR, Smith Fawzi MC, Raviola GJ, et al. Formative research on a teacher accompaniment model to promote youth mental health in Haiti: Relevance to mental health task-sharing in low-resource school settings. International Journal of Social Psychiatry. 2017;63(4):314–24.

79. Evans CL, Bazant E, Atukunda I, Williams E, Niermeyer S, Hiner C, et al. Peer-assisted learning after onsite, low-dose, high-frequency training and practice on simulators to prevent and treat postpartum hemorrhage and neonatal asphyxia: a pragmatic trial in 12 districts in Uganda. PloS one. 2018;13(12):e0207909.

80. Fabian KE, Muanido A, Cumbe VF, Manaca N, Hicks L, Weiner BJ, et al. Optimizing treatment cascades for mental healthcare in Mozambique: preliminary effectiveness of the Systems Analysis and Improvement Approach for Mental Health (SAIA-MH). Health Policy and Planning. 2020;35(10):1354–63.

81. Fils-Aimé J, Grelotti D, Thérosmé T, Kaiser B, Raviola G, Alcindor Y, et al. A mobile clinic approach to the delivery of community-based mental health services in rural Haiti. PLOS ONE. 2018;13(6).

82. Fischer EA, Jayana K, Cunningham T, Washington M, Mony P, Bradley J, et al. Nurse Mentors to Advance Quality Improvement in Primary Health Centers: Lessons From a Pilot Program in Northern Karnataka, India. Global health, science and practice. 2015;3(4):660–75.

83. Garcia M, Li M, Siril H, Hawkins C, Kaaya S, Ismail S, et al. Health-care worker engagement in HIV-related quality improvement in Dar es Salaam, Tanzania. INTERNATIONAL JOURNAL FOR QUALITY IN HEALTH CARE. 2011;23(3):231–8.

84. Gholipour K, Tabrizi J, Asghari Jafarabadi M, Iezadi S, Farshbaf N, Farzam Rahbar F, et al. Customer’s self-audit to improve the technical quality of maternity care in Tabriz: a community trial. EMHJ-Eastern Mediterranean Health Journal. 2016;22(5):309–17.

85. Gholipour K, Tabrizi JS, Asghari Jafarabadi M, Iezadi S, Mardi A. Effects of customer self-audit on the quality of maternity care in Tabriz: a cluster-randomized controlled trial. PLoS One. 2018;13(10):e0203255.

86. Ghosh R, Spindler H, Morgan MC, Cohen SR, Begum N, Gore A, et al. Diagnosis and management of postpartum hemorrhage and intrapartum asphyxia in a quality improvement initiative using nurse-mentoring and simulation in Bihar, India. Plos one. 2019;14(7):e0216654.

87. Giannitrapani K, Satija A, Ganesh A, Gamboa R, Fereydooni S, Hennings T, et al. Barriers and Facilitators of Using Quality Improvement To Foster Locally Initiated Innovation in Palliative Care Services in India. JOURNAL OF GENERAL INTERNAL MEDICINE. 2021;36(2):366–73.

88. Giessler K, Seefeld A, Montagu D, Phillips B, Mwangi J, Munson M, et al. Perspectives on implementing a quality improvement collaborative to improve person-centered care for maternal and reproductive health in Kenya. International Journal for Quality in Health Care. 2020;32(10):671–6.

89. Gilson L, Ellokor S, Lehmann U, Brady L. Organizational change and everyday health system resilience: Lessons from Cape Town, South Africa. SOCIAL SCIENCE & MEDICINE. 2020;266.

90. Gimbel S, Rustagi AS, Robinson J, Kouyate S, Coutinho J, Nduati R, et al. Evaluation of a systems analysis and improvement approach to optimize prevention of mother-to-child transmission of HIV using the consolidated framework for implementation research. JAIDS Journal of Acquired Immune Deficiency Syndromes. 2016;72:S108–16.

91. Girot EA, Enders BC, Wright J. Transforming the obstetric nursing workforce in NE Brazil through international collaboration. Journal of advanced nursing. 2005;50(6):651–60.

92. Glaser J, Hansson E, Weiss I, Wesseling C, Jakobsson K, Ekström U, et al. Preventing kidney injury among sugarcane workers: promising evidence from enhanced workplace interventions. Occupational and environmental medicine. 2020;77(8):527–34.

93. Goicolea I, Coe A, San Sebastián M, Hurtig A. Developing and sustaining adolescent-friendly health services: A multiple case study from Ecuador and Peru. GLOBAL PUBLIC HEALTH. 2017;12(8):1004–17.

94. Gomes ML, Nicida LR de A, de Oliveira DCC, Rodrigues A, Torres JA, Coutinho A da TD, et al. Care at the first postnatal hour in two hospitals of the Adequate Birth Project: qualitative analysis of experiences in two stages of the Healthy Birth research. Reproductive Health. 2022;20(Suppl 2):14.

95. Goodman C, Opwora A, Kabare M, Molyneux S. Health facility committees and facility management-exploring the nature and depth of their roles in Coast Province, Kenya. BMC health services research. 2011;11:1–12.

96. Goyet S, Rajbhandari S, Alvarez V, Bayou A, Khanal S, Pokhrel T. On-site clinical mentoring as a maternal and new-born care quality improvement method: evidence from a nurse cohort study in Nepal. BMC NURSING. 2020;19(1).

97. Grimsrud A, Sharp J, Kalombo C, Bekker L, Myer L. Implementation of community‐based adherence clubs for stable antiretroviral therapy patients in Cape Town, South Africa. Journal of the International AIDS Society. 2015;18(1):19984.

98. Gullo S, Galavotti C, Kuhlmann A, Msiska T, Hastings P, Marti C. Effects of the Community Score Card approach on reproductive health service-related outcomes in Malawi. PLOS ONE. 2020;15(5).

99. Gullo S, Galavotti C, Sebert Kuhlmann A, Msiska T, Hastings P, Marti CN. Effects of a social accountability approach, CARE’s Community Score Card, on reproductive health-related outcomes in Malawi: a cluster-randomized controlled evaluation. PLoS one. 2017;12(2):e0171316.

100. Gulzar J, Ali M, Kuroiwa C. A social marketing approach to quality improvement in family planning services: a case study from Rawalpindi, Pakistan. BIOSCIENCE TRENDS. 2008;2(1):15–21.

101. Gupta A, Agrawal R, Gupt A, Guleri R, Bajpayee D, Joshi N, et al. Systems E-approach for women at risk (SEWA)—A digital health solution for detection of high-risk pregnancies. Journal of family medicine and primary care. 2021;10(10):3712–9.

102. Hall H, Mahmood M, Sitaing M, Aines P, Cant R, Crawford K. The PNG Midwifery Leadership Buddy Program: An evaluation. WOMEN AND BIRTH. 2023;36(5):e536–43.

103. Henry EG, Thea DM, Hamer DH, DeJong W, Musokotwane K, Chibwe K, et al. The impact of a multi-level maternal health programme on facility delivery and capacity for emergency obstetric care in Zambia. Global public health. 2018;13(10):1481–94.

104. Hill Z, Keraga D, Alemayehu A, Schellenberg J, Magge H, Estifanos A. “The objective was about not blaming one another”: a qualitative study to explore how collaboration is experienced within quality improvement collaboratives in Ethiopia. HEALTH RESEARCH POLICY AND SYSTEMS. 2023;21(1).

105. Hirschhorn L, Semrau K, Kodkany B, Churchill R, Kapoor A, Spector J, et al. Learning before leaping: integration of an adaptive study design process prior to initiation of BetterBirth, a large-scale randomized controlled trial in Uttar Pradesh, India. IMPLEMENTATION SCIENCE. 2015;10.

106. Ho LS, Labrecque G, Batonon I, Salsi V, Ratnayake R. Effects of a community scorecard on improving the local health system in Eastern Democratic Republic of Congo: qualitative evidence using the most significant change technique. Conflict and Health. 2015;9:1–11.

107. Hofman J, Mohammed H. Experiences with facility-based maternal death reviews in northern Nigeria. INTERNATIONAL JOURNAL OF GYNECOLOGY & OBSTETRICS. 2014;126(2):111–4.

108. Hofmeyr G, Ryan R, Mussa A, Bame B, Malima S, Moloi T, et al. Improving postpregnancy contraceptive method choice and long-acting reversible contraception provision in Botswana: a quality improvement pilot. BMJ SEXUAL & REPRODUCTIVE HEALTH. 2023;49(4):293–7.

109. Horwood C, Butler L, Barker P, Phakathi S, Haskins L, Grant M, et al. A continuous quality improvement intervention to improve the effectiveness of community health workers providing care to mothers and children: a cluster randomised controlled trial in South Africa. Human resources for health. 2017;15:1–11.

110. Ibiloye O, Decroo T, Eyona N, Eze P, Agada P. Characteristics and early clinical outcomes of key populations attending comprehensive community-based HIV care: Experiences from Nasarawa State, Nigeria. PLoS One. 2018;13(12):e0209477.

111. Inzunza M, Carlsson N. Crime prevention in Colombia: A pilot study. INTERNATIONAL JOURNAL OF COMPARATIVE AND APPLIED CRIMINAL JUSTICE. 2023;47(3):279–98.

112. Iyengar K, Jain M, Thomas S, Dashora K, Liu W, Saini P, et al. Adherence to evidence based care practices for childbirth before and after a quality improvement intervention in health facilities of Rajasthan, India. BMC pregnancy and childbirth. 2014;14:1–12.

113. Izudi J, Mugenyi J, Mugabekazi M, Muwanika B, Spector V, Katawera A, et al. Retention of HIV-Positive Adolescents in Care: A Quality Improvement Intervention in Mid-Western Uganda. BIOMED RESEARCH INTERNATIONAL. 2018;2018.

114. Izudi J, Owoko HO, Bagayoko M, Kadengye D. Experiences of mothers and health workers with MomCare and SafeCare bundles in Kenya and Tanzania: A qualitative evaluation. PloS one. 2023;18(11):e0294536.

115. Jamisse L, Songane F, Libombo A, Bique C, Faúndes A. Reducing maternal mortality in Mozambique: challenges, failures, successes and lessons learned. International Journal of Gynecology & Obstetrics. 2004;85(2):203–12.

116. Jaribu J, Penfold S, Manzi F, Schellenberg J, Pfeiffer C. Improving institutional childbirth services in rural Southern Tanzania: a qualitative study of healthcare workers’ perspective. BMJ open. 2016;6(9):e010317.

117. Jassani K, Essani RR, Abbas SNH. Quality, Safety and Patient Centered Care--A Dream Come True in the Mountains of Northern Pakistan. An Award winning project of “2015 Quality, Safety & Patient Centered Care Award” at, Chicago USA. World hospitals and health services : the official journal of the International Hospital Federation. 2016;52(1):17–20.

118. Jassani K, Essani RRA, Abbas N, Ahmed R. Quality improvement initiatives by Aga Khan Health Service in the mountains of northern Pakistan. World hospitals and health services : the official journal of the International Hospital Federation. 2015;51(1):32–5.

119. Jayanna K, Bradley J, Mony P, Cunningham T, Washington M, Bhat S, et al. Effectiveness of onsite nurse mentoring in improving quality of institutional births in the primary health centres of high priority districts of Karnataka, South India: a cluster randomized trial. PloS one. 2016;11(9):e0161957.

120. Kabongo L, Gass J, Kivondo B, Kara N, Semrau K, Hirschhorn LR. Implementing the WHO Safe Childbirth Checklist: lessons learnt on a quality improvement initiative to improve mother and newborn care at Gobabis District Hospital, Namibia. BMJ open quality. 2017;6(2):e000145.

121. Kane S, Dayal P, Mahapatra T, Kumar S, Bhasin S, Gore A, et al. Enabling change in public health services: Insights from the implementation of nurse mentoring interventions to improve quality of obstetric and newborn care in two North Indian states. Gates open research. 2021;4:61.

122. Kapologwe NA, Kabengula JS, Msuya SE. Perceived barriers and attitudes of health care providers towards Provider-Initiated HIV Testing and Counseling in Mbeya region, southern highland zone of Tanzania. Pan African Medical Journal. 2011;8(1).

123. Kasenga F, Byass P, Emmelin M, Hurtig AK. The implications of policy changes on the uptake of a PMTCT programme in rural Malawi: first three years of experience. Global health action. 2009;2(1):1883.

124. Kassa G, Dougherty G, Madevu-Matson C, Egesimba G, Sartie K, Akinjeji A, et al. Improving inpatient provider-initiated HIV testing and counseling in Sierra Leone. PLoS One. 2020;15(7):e0236358.

125. Kayongo M, Butera J, Mboninyibuka D, Nyiransabimana B, Ntezimana A, Mukangamuje V. Improving availability of EmOC services in Rwanda—CARE’s experiences and lessons learned at Kabgayi Referral Hospital. International Journal of Gynecology & Obstetrics. 2006;92(3):291–8.

126. Kayongo M, Esquiche E, Luna M, Frias G, Vega-Centeno L, Bailey P. Strengthening emergency obstetric care in Ayacucho, Peru. International Journal of Gynecology & Obstetrics. 2006;92(3):299–307.

127. Kayongo M, Rubardt M, Butera J, Abdullah M, Mboninyibuka D, Madili M. Making EmOC a reality—CARE’s experiences in areas of high maternal mortality in Africa. International Journal of Gynecology & Obstetrics. 2006;92(3):308–19.

128. Khurram Azmat S, Tasneem Shaikh B, Hameed W, Mustafa G, Hussain W, Asghar J, et al. Impact of social franchising on contraceptive use when complemented by vouchers: a quasi-experimental study in rural Pakistan. PloS one. 2013;8(9):e74260.

129. Kim MH, Ahmed S, Buck WC, Preidis GA, Hosseinipour MC, Bhalakia A, et al. The Tingathe programme: a pilot intervention using community health workers to create a continuum of care in the prevention of mother to child transmission of HIV (PMTCT) cascade of services in Malawi. Journal of the International AIDS Society. 2012;15:17389.

130. Kim YM, Banda J, Kanjipite W, Sarkar S, Bazant E, Hiner C, et al. Improving performance of Zambia Defence Force antiretroviral therapy providers: evaluation of a standards-based approach. Global Health: Science and Practice. 2013;1(2):213–27.

131. Kim YM, Chilila M, Shasulwe H, Banda J, Kanjipite W, Sarkar S, et al. Evaluation of a quality improvement intervention to prevent mother-to-child transmission of HIV (PMTCT) at Zambia defence force facilities. BMC health services research. 2013;13:1–14.

132. Kosgei RJ, Lubano KM, Shen C, Wools-Kaloustian KK, Musick BS, Siika AM, et al. Impact of integrated family planning and HIV care services on contraceptive use and pregnancy outcomes: a retrospective cohort study. JAIDS Journal of Acquired Immune Deficiency Syndromes. 2011;58(5):e121–6.

133. Kruk ME, Vail D, Austin-Evelyn K, Atuyambe L, Greeson D, Grépin KA, et al. Evaluation of a maternal health program in Uganda and Zambia finds mixed results on quality of care and satisfaction. Health Affairs. 2016;35(3):510–9.

134. Kujawski SA, Freedman LP, Ramsey K, Mbaruku G, Mbuyita S, Moyo W, et al. Community and health system intervention to reduce disrespect and abuse during childbirth in Tanga region, Tanzania: a comparative before-and-after study. PLoS medicine. 2017;14(7):e1002341.

135. Kulkarni V, Vohra N, Sharma S, Nair N. Walking the tightrope: Gender inclusion as organizational change. Journal of Organizational Change Management. 2021;34(1):106–20.

136. Kumar A, Lakshminarayanan D, Joshi N, Vaid S, Bhoi S, Deorari A. Triaging the triage: reducing waiting time to triage in the emergency department at a tertiary care hospital in New Delhi, India. EMERGENCY MEDICINE JOURNAL. 2019;36(9):558-+.

137. Kumar M, Schulte-Hillen C, De Plecker E, Van Haver A, Marques S, Daly M, et al. Catalyst for change: Lessons learned from overcoming barriers to providing safe abortion care in Medecins Sans Frontieres projects. PERSPECTIVES ON SEXUAL AND REPRODUCTIVE HEALTH. 2022;

138. Kumar S, Yadav V, Balasubramaniam S, Jain Y, Joshi CS, Saran K, et al. Effectiveness of the WHO SCC on improving adherence to essential practices during childbirth, in resource constrained settings. BMC pregnancy and childbirth. 2016;16:1–11.

139. LaCourse SM, Chester FM, Matoga M, Munthali C, Nsona D, Haac B, et al. Implementation and operational research: implementation of routine counselor-initiated Opt-Out HIV testing on the adult medical ward at Kamuzu Central Hospital, Lilongwe, Malawi. JAIDS Journal of Acquired Immune Deficiency Syndromes. 2015;69(1):e31–5.

140. Landes M, Thompson C, Mwinjiwa E, Thaulo E, Gondwe C, Akello H, et al. Task shifting of triage to peer expert informal care providers at a tertiary referral HIV clinic in Malawi: a cross-sectional operational evaluation. BMC HEALTH SERVICES RESEARCH. 2017;17.

141. Larson E, Gage A, Mbaruku G, Mbatia R, Haneuse S, Kruk M. Effect of a maternal and newborn health system quality improvement project on the use of facilities for childbirth: a cluster-randomised study in rural Tanzania. TROPICAL MEDICINE & INTERNATIONAL HEALTH. 2019;24(5):636–46.

142. Laterra A, Callahan T, Msiska T, Woelk G, Chowdhary P, Gullo S, et al. Bringing women’s voices to PMTCT CARE: adapting CARE’s Community Score Card© to engage women living with HIV to build quality health systems in Malawi. BMC HEALTH SERVICES RESEARCH. 2020;20(1).

143. Liambila W, Askew I, Mwangi J, Ayisi R, Kibaru J, Mullick S. Feasibility and effectiveness of integrating provider-initiated testing and counselling within family planning services in Kenya. Aids. 2009;23:S115–21.

144. Limato R, Tumbelaka P, Ahmed R, Nasir S, Syafruddin D, Ormel H, et al. What factors do make quality improvement work in primary health care? Experiences of maternal health quality improvement teams in three Puskesmas in Indonesia. PloS one. 2019;14(12):e0226804.

145. Liu L, Christie S, Munsamy M, Roberts P, Pillay M, Shenoi SV, et al. Expansion of a national differentiated service delivery model to support people living with HIV and other chronic conditions in South Africa: a descriptive analysis. BMC health services research. 2021;21:1–8.

146. Liu Y, Zhu W, Le S, Wu W, Huang Q, Cheng W. Using healthcare failure mode and effect analysis as a method of vaginal birth after caesarean section management. JOURNAL OF CLINICAL NURSING. 2020;29(1–2):130–8.

147. Lodenstein E, Mafuta E, Kpatchavi AC, Servais J, Dieleman M, Broerse JE, et al. Social accountability in primary health care in West and Central Africa: exploring the role of health facility committees. BMC health services research. 2017;17:1–15.

148. Ludwick T, Endriyas M, Morgan A, Kane S, Kelaher M, McPake B. Challenges in Implementing Community-Based Healthcare Teams in a Low-Income Country Context: Lessons From Ethiopia’s Family Health Teams. INTERNATIONAL JOURNAL OF HEALTH POLICY AND MANAGEMENT. 2021;

149. Madede T, Sidat M, McAuliffe E, Patricio SR, Uduma O, Galligan M, et al. The impact of a supportive supervision intervention on health workers in Niassa, Mozambique: a cluster-controlled trial. Human resources for health. 2017;15:1–11.

150. Magar V, Kambou S. Sexuality and gender: change through reflection and action. CRITICAL PUBLIC HEALTH. 2012;22(1):85–98.

151. Maharaj P, Cleland J. Integration of sexual and reproductive health services in KwaZulu-Natal, South Africa. Health policy and planning. 2005;20(5):310–8.

152. Mash BJ, Mayers P, Conradie H, Orayn A, Kuiper M, Marais J. How to manage organisational change and create practice teams: experiences of a South African primary care health centre. Education for health (Abingdon, England). 2008;21(2):132.

153. Mate K, Ngubane G, Barker P. A quality improvement model for the rapid scale-up of a program to prevent mother-to-child HIV transmission in South Africa. INTERNATIONAL JOURNAL FOR QUALITY IN HEALTH CARE. 2013;25(4):373–80.

154. Mathews C, Guttmacher SJ, Flisher AJ, Mtshizana YY, Nelson T, McCarthy J, et al. The quality of HIV testing services for adolescents in Cape Town, South Africa: do adolescent-friendly services make a difference? Journal of Adolescent Health. 2009;44(2):188–90.

155. Mayhew SH, Lush L, Cleland J, Walt G. Implementing the integration of component services for reproductive health. 2000;

156. McBride K, Parent J, Mmanga K, Chivwala M, Nyirenda M, Schooley A, et al. ART Adherence Among Malawian Youth Enrolled in Teen Clubs: A Retrospective Chart Review. AIDS AND BEHAVIOR. 2019;23(9):2629–33.

157. McCarraher DR, Vance G, Gwarzo U, Taylor D, Chabikuli ON. Changes in contraceptive use following integration of family planning into ART services in Cross River State, Nigeria. Studies in Family Planning. 2011;42(4):283–90.

158. McCarthy EA, Subramaniam HL, Prust ML, Prescott MR, Mpasela F, Mwango A, et al. Quality improvement intervention to increase adherence to ART prescription policy at HIV treatment clinics in Lusaka, Zambia: A cluster randomized trial. PloS one. 2017;12(4):e0175534.

159. McCollum ED, Preidis GA, Kabue MM, Singogo EB, Mwansambo C, Kazembe PN, et al. Task shifting routine inpatient pediatric HIV testing improves program outcomes in urban Malawi: a retrospective observational study. PloS one. 2010;5(3):e9626.

160. McGivern G, Nzinga J, English M. “Pastoral practices” for quality improvement in a Kenyan clinical network. SOCIAL SCIENCE & MEDICINE. 2017;195:115–22.

161. McLean KE, Kaiser BN, Hagaman AK, Wagenaar BH, Therosme TP, Kohrt BA. Task sharing in rural Haiti: qualitative assessment of a brief, structured training with and without apprenticeship supervision for community health workers. Intervention (Amstelveen, Netherlands). 2015;13(2):135.

162. Mengistu B, Alemu H, Kassa M, Zelalem M, Abate M, Bitewulign B, et al. An innovative intervention to improve respectful maternity care in three Districts in Ethiopia. BMC PREGNANCY AND CHILDBIRTH. 2021;21(1).

163. Meuwissen LE, Gorter AC, Kester AD, Knottnerus J. Can a comprehensive voucher programme prompt changes in doctors’ knowledge, attitudes and practices related to sexual and reproductive health care for adolescents? A case study from Latin America. Tropical Medicine & International Health. 2006;11(6):889–98.

164. Meuwissen LE, Gorter AC, Kester AD, Knottnerus JA. Does a competitive voucher program for adolescents improve the quality of reproductive health care? A simulated patient study in Nicaragua. BMC Public Health. 2006;6:1–9.

165. Meuwissen LE, Gorter AC, Knottnerus JA. Perceived quality of reproductive care for girls in a competitive voucher programme. A quasi-experimental intervention study, Managua, Nicaragua. International Journal for Quality in Health Care. 2006;18(1):35–42.

166. Molyneux E, Ahmad S, Robertson A. Improved triage and emergency care for children reduces inpatient mortality in a resource-constrained setting. Bulletin of the World Health Organization. 2006;84(4):314–9.

167. Mudhune S, Phiri SC, Prescott MR, McCarthy EA, Banda A, Haimbe P, et al. Improving the quality of childbirth services in Zambia through introduction of the Safe Childbirth Checklist and systems-focused mentorship. Plos one. 2020;15(12):e0244310.

168. Mugore S, Kassouta NTK, Sebikali B, Lundstrom L, Saad A. Improving the Quality of Postabortion Care Services in Togo Increased Uptake of Contraception. Global health, science and practice. 2016;4(3):495–505.

169. Mukinda F, Van Belle S, Schneider H. Local Dynamics of Collaboration for Maternal, Newborn and Child Health: A Social Network Analysis of Healthcare Providers and Their Managers in Gert Sibande District, South Africa. INTERNATIONAL JOURNAL OF HEALTH POLICY AND MANAGEMENT. 2021;

170. Mwango LK, Stafford KA, Blanco NC, Lavoie M, Mujansi M, Nyirongo N, et al. Index and targeted community‐based testing to optimize HIV case finding and ART linkage among men in Zambia. Journal of the International AIDS Society. 2020;23:e25520.

171. Nahimana E, McBain R, Manzi A, Iyer H, Uwingabiye A, Gupta N, et al. Race to the Top: evaluation of a novel performance-based financing initiative to promote healthcare delivery in rural Rwanda. GLOBAL HEALTH ACTION. 2016;9.

172. Naidoo M, Moodley J, Gathiram P, Sartorius B. The impact of a modified World Health Organization surgical safety checklist on maternal outcomes in a South African setting: A stratified cluster-randomised controlled trial. South African Medical Journal. 2017;107(3):248–57.

173. Naikoba S, Senjovu KD, Mugabe P, McCarthy CF, Riley PL, Kadengye DT, et al. Improved HIV and TB knowledge and competence among mid-level providers in a cluster-randomized trial of one-on-one mentorship for task shifting. JAIDS Journal of Acquired Immune Deficiency Syndromes. 2017;75(5):e120–7.

174. Ngo TD, Nuccio O, Pereira SK, Footman K, Reiss K. Evaluating a LARC expansion program in 14 sub-Saharan African countries: a service delivery model for meeting FP2020 goals. Maternal and child health journal. 2017;21:1734–43.

175. Nyamtema AS, de Jong AB, Urassa DP, Van Roosmalen J. Using audit to enhance quality of maternity care in resource limited countries: lessons learnt from rural Tanzania. BMC pregnancy and childbirth. 2011;11:1–8.

176. Nyamtema AS, Mwakatundu N, Dominico S, Mohamed H, Pemba S, Rumanyika R, et al. Enhancing maternal and perinatal health in under-served remote areas in sub-Saharan Africa: a Tanzanian model. PLoS One. 2016;11(3):e0151419.

177. Obare F, Warren C, Njuki R, Abuya T, Sunday J, Askew I, et al. Community-level impact of the reproductive health vouchers programme on service utilization in Kenya. Health Policy and Planning. 2013;28(2):165–75.

178. Obua C, Kayiwa J, Waako P, Tomson G, Balidawa H, Chalker J, et al. Improving adherence to antiretroviral treatment in Uganda with a low-resource facility-based intervention. Global health action. 2014;7(1):24198.

179. Oladele EA, Badejo OA, Obanubi C, Okechukwu EF, James E, Owhonda G, et al. Bridging the HIV treatment gap in Nigeria: examining community antiretroviral treatment models. Journal of the International AIDS Society. 2018;21(4):e25108.

180. Onyango D, Tumlinson K, Chung S, Bullington B, Gakii C, Senderowicz L. Evaluating the feasibility of the Community Score Card and subsequent contraceptive behavior in Kisumu, Kenya. BMC PUBLIC HEALTH. 2022;22(1).

181. Orengo-Aguayo R, Stewart R, Villalobos B, Rodriguez J, Dueweke A, de Arellano M, et al. Listen, Don’t Tell: Partnership and Adaptation to Implement Trauma-Focused Cognitive Behavioral Therapy in Low-Resourced Settings. AMERICAN PSYCHOLOGIST. 2020;75(8):1158–74.

182. Orobaton N, Nsabagasani X, Ekochu E, Oki J, Kironde S, Lippeveld T. Promoting unity of purpose in district health service delivery in Uganda through partnerships, trust building and evidence-based decision-making. Education for health (Abingdon, England). 2007;20(2):58.

183. Pacheco-Zenteno F, Glaser J, Jakobsson K, Weiss I, Arias-Monge E, Gyllensten K. The Prevention of Occupational Heat Stress in Sugarcane Workers in Nicaragua-An Interpretative Phenomenological Analysis. FRONTIERS IN PUBLIC HEALTH. 2021;9.

184. Pallangyo E, Mbekenga C, Olsson P, Eriksson L, Bergström A. Implementation of a facilitation intervention to improve postpartum care in a low-resource suburb of Dar es Salaam, Tanzania. IMPLEMENTATION SCIENCE. 2018;13.

185. Pallangyo E, Mbekenga C, Olsson P, Rubertsson C, Källestål C. Improved postpartum care after a participatory facilitation intervention in Dar es Salaam, Tanzania: a mixed method evaluation. Global health action. 2017;10(1):1295697.

186. Patabendige M, Senanayake H. Implementation of the WHO safe childbirth checklist program at a tertiary care setting in Sri Lanka: a developing country experience. BMC pregnancy and childbirth. 2015;15:1–6.

187. Pearson CR, Micek MA, Simoni JM, Hoff PD, Matediana E, Martin DP, et al. Randomized control trial of peer-delivered, modified directly observed therapy for HAART in Mozambique. JAIDS Journal of Acquired Immune Deficiency Syndromes. 2007;46(2):238–44.

188. Petersen I, van Rensburg A, Kigozi F, Semrau M, Hanlon C, Abdulmalik J, et al. Scaling up integrated primary mental health in six low- and middle-income countries: obstacles, synergies and implications for systems reform. BJPSYCH OPEN. 2019;5(5).

189. Phiri S, Feldacker C, Chaweza T, Mlundira L, Tweya H, Speight C, et al. Integrating reproductive health services into HIV care: strategies for successful implementation in a low-resource HIV clinic in Lilongwe, Malawi. The journal of family planning and reproductive health care. 2016;42(1):17–23.

190. Prasad N, Mwakatundu N, Dominico S, Masako P, Mongo W, Mwanshemele Y, et al. Improving maternal and reproductive health in Kigoma, Tanzania: a 13-year initiative. Global Health: Science and Practice. 2022;10(2).

191. Rabkin M, Achwoka D, Akoth S, Boccanera R, Kimani M, Leting I, et al. Improving utilization of HIV viral load test results using a quality improvement collaborative in Western Kenya. Journal of the Association of Nurses in AIDS Care. 2020;31(5):566–73.

192. Ramaswamy R, Kallam B, Kopic D, Pujic B, Owen M. Global health partnerships: building multinational collaborations to achieve lasting improvements in maternal and neonatal health. GLOBALIZATION AND HEALTH. 2016;12.

193. Ramiro LS, Castillo FA, Tan-Torres T, Torres CE, Tayag JG, Talampas RG, et al. Community participation in local health boards in a decentralized setting: cases from the Philippines. Health policy and planning. 2001;61–9.

194. Rasheed M, Hussain A, Hashwani A, Kedzierski J, Hasan B. Implementation evaluation of a leadership development intervention for improved family experience in a private paediatric care hospital, Pakistan. BMC HEALTH SERVICES RESEARCH. 2022;22(1).

195. Rasheed MA, Bharuchi V, Mughis W, Hussain A. Development and feasibility testing of a play-based psychosocial intervention for reduced patient stress in a pediatric care setting: experiences from Pakistan. Pilot and feasibility studies. 2021;7:1–13.

196. Rasheed MA, Kedzierski JT, Hasan BS. Improved family experience outcomes in a pediatric hospital in Pakistan: mentoring, human-centered practice, and theory of change. NEJM Catalyst Innovations in Care Delivery. 2021;2(7).

197. Rasschaert F, Telfer B, Lessitala F, Decroo T, Remartinez D, Biot M, et al. A qualitative assessment of a community antiretroviral therapy group model in Tete, Mozambique. PloS one. 2014;9(3):e91544.

198. Ratcliffe H, Sando D, Lyatuu G, Emil F, Mwanyika-Sando M, Chalamilla G, et al. Mitigating disrespect and abuse during childbirth in Tanzania: an exploratory study of the effects of two facility-based interventions in a large public hospital. REPRODUCTIVE HEALTH. 2016;13.

199. Rawlins BJ, Kim YM, Rozario AM, Bazant E, Rashidi T, Bandazi SN, et al. Reproductive health services in Malawi: an evaluation of a quality improvement intervention. Midwifery. 2013;29(1):53–9.

200. Renggli S, Mayumana I, Mboya D, Charles C, Mshana C, Kessy F, et al. Towards improved health service quality in Tanzania: contribution of a supportive supervision approach to increased quality of primary healthcare. BMC HEALTH SERVICES RESEARCH. 2019;19(1).

201. Renner HJ, Makobu NW, Mbugua S, Kamiru WK, Oluoch D, Donelson A, et al. “I Am Now Five Steps Ahead”: How Co-design Platforms Sustain Kenyan Community Health Volunteer Engagement. Progress in community health partnerships : research, education, and action. 2023;17(3):419–27.

202. Rice H, Lou-Meda R, Saxton A, Johnston B, Ramirez C, Mendez S, et al. Building a safety culture in global health: lessons from Guatemala. BMJ GLOBAL HEALTH. 2018;3(2).

203. Rodrigues A, de Oliveira D, Gomes M, Nicida L, Torres J, Coutinho A, et al. Women’s voice on changes in childbirth care practices: a qualitative approach to women’s experiences in Brazilian private hospitals participating in the Adequate Childbirth Project. REPRODUCTIVE HEALTH. 2023;20(SUPPL 2).

204. Rustagi AS, Gimbel S, Nduati R, de Fatima Cuembelo M, Wasserheit JN, Farquhar C, et al. Impact of a systems engineering intervention on PMTCT service delivery in Cote d’Ivoire, Kenya, Mozambique: a cluster randomized trial. Journal of acquired immune deficiency syndromes (1999). 2016;72(3):e68.

205. Sam-Agudu NA, Ramadhani HO, Isah C, Anaba U, Erekaha S, Fan-Osuala C, et al. The impact of structured mentor mother programs on 6-month postpartum retention and viral suppression among HIV-positive women in rural Nigeria: a prospective paired cohort study. JAIDS Journal of Acquired Immune Deficiency Syndromes. 2017;75:S173–81.

206. Sanjana P, Torpey K, Schwarzwalder A, Simumba C, Kasonde P, Nyirenda L, et al. Task-shifting HIV counselling and testing services in Zambia: the role of lay counsellors. Human resources for health. 2009;7:1–7.

207. Santos C, Diante D, Baptista A, Matediane E, Bique C, Bailey P. Improving emergency obstetric care in Mozambique: The story of Sofala. INTERNATIONAL JOURNAL OF GYNECOLOGY & OBSTETRICS. 2006;94(2):190–201.

208. Sarin E, Kole SK, Patel R, Sooden A, Kharwal S, Singh R, et al. Evaluation of a quality improvement intervention for obstetric and neonatal care in selected public health facilities across six states of India. BMC Pregnancy and Childbirth. 2017;17:1–10.

209. Sarkar A, Selvam S, Chawla R, Jindal S, Thakur V. Addressing the high prevalence of uterine fundal pressure in low-middle income country during vaginal delivery through a quality improvement initiative: Road to respectful maternity care. JOURNAL OF OBSTETRICS AND GYNAECOLOGY RESEARCH. 2023;49(1):194–200.

210. Sarkar A, Wadhawan I, Raj A, Nagabhushana P, Singh P. Improving the Acceptance Rate of Centchroman As a Postpartum Contraceptive Through a Quality Improvement Initiative. CUREUS JOURNAL OF MEDICAL SCIENCE. 2022;14(9).

211. Sarwal T, Sarwal Y, Tyagi S, Sarwal R. Healthcare providers perceptions regarding the presence of Birth Companion during childbirth at a tertiary care hospital in India. BMC PREGNANCY AND CHILDBIRTH. 2023;23(1).

212. Satija A, Lorenz KA, Spruijt O, Ganesh A, Singh N, Connell NB, et al. Quality Improvement in Itself Changes Your Thinking : Lessons From Disseminating Quality Improvement Methods Through a Multisite International Collaborative Palliative Care Project in India. JCO global oncology. 2022;8:e2200147.

213. Saving Mothers Giving Life Wor, Ngoma-Hazemba A, Hamomba L, Silumbwe A, Munakampe M, Soud F. Community Perspectives of a 3-Delays Model Intervention: A Qualitative Evaluation of Saving Mothers, Giving Life in Zambia. GLOBAL HEALTH-SCIENCE AND PRACTICE. 2019;7:S139–50.

214. Schneider H, George A, Mukinda F, Tabana H. District governance and improved maternal, neonatal and child health in South Africa: pathways of change. Health Systems & Reform. 2020;6(1):e1669943.

215. Scoular S, Malhotra J, Valdez C. Development and implementation of a remotely precepted, interprofessional advanced pharmacy practice experience in rural Guatemala. CURRENTS IN PHARMACY TEACHING AND LEARNING. 2020;12(8):1014–20.

216. Selke HM, Kimaiyo S, Sidle JE, Vedanthan R, Tierney WM, Shen C, et al. Task-shifting of antiretroviral delivery from health care workers to persons living with HIV/AIDS: clinical outcomes of a community-based program in Kenya. JAIDS Journal of Acquired Immune Deficiency Syndromes. 2010;55(4):483–90.

217. Senanayake H, Wijesinghe RD, Nayar KR. Is the policy of allowing a female labor companion feasible in developing countries? Results from a cross sectional study among Sri Lankan practitioners. BMC pregnancy and childbirth. 2017;17:1–6.

218. Serbanescu F, Goldberg HI, Danel I, Wuhib T, Marum L, Obiero W, et al. Rapid reduction of maternal mortality in Uganda and Zambia through the saving mothers, giving life initiative: results of year 1 evaluation. BMC pregnancy and childbirth. 2017;17:1–14.

219. Seshadri S, Parab S, Kotte S, Latha N, Subbiah K. Decentralization and decision space in the health sector: a case study from Karnataka, India. HEALTH POLICY AND PLANNING. 2016;31(2):171–81.

220. Sherr K, Ásbjörnsdóttir K, Crocker J, Coutinho J, de Fatima Cuembelo M, Tavede E, et al. Scaling-up the Systems Analysis and Improvement Approach for prevention of mother-to-child HIV transmission in Mozambique (SAIA-SCALE): a stepped-wedge cluster randomized trial. Implementation Science. 2019;14:1–13.

221. Shimkhada R, Peabody JW, Quimbo SA, Solon O. The Quality Improvement Demonstration Study: an example of evidence-based policy-making in practice. Health research policy and systems. 2008;6:5.

222. Shukla A, Khanna R, Jadhav N. Using community-based evidence for decentralized health planning: insights from Maharashtra, India. Health Policy and Planning. 2018;33(1):e34–45.

223. Sibley L, Tesfaye S, Desta B, Frew A, Kebede A, Mohammed H, et al. Improving Maternal and Newborn Health Care Delivery in Rural Amhara and Oromiya Regions of Ethiopia Through the Maternal and Newborn Health in Ethiopia Partnership. JOURNAL OF MIDWIFERY & WOMENS HEALTH. 2014;59:S6–20.

224. Silverstein A, Benson A, Gates C, Nguyen D. Global community of practice: A means for capacity and community strengthening for health professionals in low- and middle-income countries. JOURNAL OF GLOBAL HEALTH. 2022;12.

225. Singh K, Brodish P, Speizer I, Barker P, Amenga-Etego I, Dasoberi I, et al. Can a quality improvement project impact maternal and child health outcomes at scale in northern Ghana? Health research policy and systems. 2016;14:1–13.

226. Singh K, Speizer I, Handa S, Boadu RO, Atinbire S, Barker PM, et al. Impact evaluation of a quality improvement intervention on maternal and child health outcomes in Northern Ghana: early assessment of a national scale-up project. International journal for quality in health care. 2013;25(5):477–87.

227. Singh S, Kannuri N, Mishra A, Gaikwad L, Shukla R, Tyagi M, et al. Evaluation of Dakshata, a scale-up WHO SCC and mentoring-based program, for improving quality of intrapartum care in public sector in Rajasthan, India: repeated mixed-methods surveys. ARCHIVES OF PUBLIC HEALTH. 2023;81(1).

228. Sitienei J, Manderson L, Nangami M. Community participation in the collaborative governance of primary health care facilities, Uasin Gishu County, Kenya. PLOS ONE. 2021;16(3).

229. Spector JM, Agrawal P, Kodkany B, Lipsitz S, Lashoher A, Dziekan G, et al. Improving quality of care for maternal and newborn health: prospective pilot study of the WHO safe childbirth checklist program. PloS one. 2012;7(5):e35151.

230. Srofenyoh E, Kassebaum N, Goodman D, Olufolabi A, Owen M. Measuring the impact of a quality improvement collaboration to decrease maternal mortality in a Ghanaian regional hospital. INTERNATIONAL JOURNAL OF GYNECOLOGY & OBSTETRICS. 2016;134(2):181–5.

231. Srofenyoh E, Ivester T, Engmann C, Olufolabi A, Bookman L, Owen M. Advancing obstetric and neonatal care in a regional hospital in Ghana via continuous quality improvement. International Journal of Gynecology & Obstetrics. 2012;116(1):17–21.

232. Stanback J, Griffey S, Lynam P, Ruto C, Cummings S. Improving adherence to family planning guidelines in Kenya: an experiment. International journal for quality in health care. 2007;19(2):68–73.

233. Stover KE, Tesfaye S, Frew AH, Mohammed H, Barry D, Alamineh L, et al. Building district-level capacity for continuous improvement in maternal and newborn health. Journal of midwifery & women’s health. 2014;59 Suppl 1:S91–100.

234. Suarez‐Balcazar Y, Hammel J, Mayo L, Inwald S, Sen S. Innovation in global collaborations: From student placement to mutually beneficial exchanges. Occupational Therapy International. 2013;20(2):94–101.

235. Suh S, Moreira P, Ly M. Improving quality of reproductive health care in Senegal through formative supervision: results from four districts. Human resources for health. 2007;5:1–12.

236. Swaans K, Broerse J, Meincke M, Mudhara M, Bunders J. Promoting food security and well-being among poor and HIV/AIDS affected households: Lessons from an interactive and integrated approach. Evaluation and Program Planning. 2009;32(1):31–42.

237. Teng-Calleja M, Hechanova M, Alampay R, Canoy N, Franco E, Alampay E. Transformation in Philippine local government. LOCAL GOVERNMENT STUDIES. 2017;43(1):64–88.

238. Tenthani L, Cataldo F, Chan AK, Bedell R, Martiniuk AL, van Lettow M. Involving expert patients in antiretroviral treatment provision in a tertiary referral hospital HIV clinic in Malawi. BMC health services research. 2012;12:1–8.

239. Torres JA, Leite TH, Fonseca TCO, Domingues RMSM, Figueiró AC, Pereira APE, et al. An implementation analysis of a quality improvement project to reduce cesarean section in Brazilian private hospitals. Reproductive Health. 2022;20(Suppl 2):190.

240. Tsondai PR, Wilkinson LS, Grimsrud A, Mdlalo PT, Ullauri A, Boulle A. High rates of retention and viral suppression in the scale‐up of antiretroviral therapy adherence clubs in Cape Town, South Africa. Journal of the International AIDS Society. 2017;20:21649.

241. van den Akker T, Bemelmans M, Ford N, Jemu M, Diggle E, Scheffer S, et al. HIV care need not hamper maternity care: a descriptive analysis of integration of services in rural Malawi. BJOG: An International Journal of Obstetrics & Gynaecology. 2012;119(4):431–8.

242. Vargas I, Eguiguren P, Mogollón-Pérez A, Samico I, Bertolotto F, López-Vázquez J, et al. Can care coordination across levels be improved through the implementation of participatory action research interventions? Outcomes and conditions for sustaining changes in five Latin American countries. BMC HEALTH SERVICES RESEARCH. 2020;20(1).

243. Vo BN, Cohen CR, Smith RM, Bukusi EA, Onono MA, Schwartz K, et al. Patient satisfaction with integrated HIV and antenatal care services in rural Kenya. AIDS care. 2012;24(11):1442–7.

244. Wagner A, Mugo C, Bluemer-Miroite S, Mutiti P, Wamalwa D, Bukusi D, et al. Continuous quality improvement intervention for adolescent and young adult HIV testing services in Kenya improves HIV knowledge. AIDS. 2017;31:S243–52.

245. Wagner AD, Augusto O, Njuguna IN, Gaitho D, Mburu N, Oluoch G, et al. Systems Analysis and Improvement Approach to optimize the pediatric and adolescent HIV Cascade (SAIA-PEDS): a pilot study. Implementation Science Communications. 2022;3(1):49.

246. Wagner G, Ryan G, Taylor S. Formative evaluation of antiretroviral therapy scale-up efficiency in sub-Saharan Africa. AIDS patient care and STDs. 2007;21(11):871–88.

247. Waiswa P, Manzi F, Mbaruku G, Rowe A, Marx M, Tomson G, et al. Effects of the EQUIP quasi-experimental study testing a collaborative quality improvement approach for maternal and newborn health care in Tanzania and Uganda. Implementation Science. 2017;12:1–18.

248. Washington M, Jayanna K, Bhat S, Thomas A, Rao S, Perumal G, et al. Nurse mentor training program to improve quality of maternal and newborn care at primary health centres: process evaluation. Open Journal of Nursing. 2016;6(6):458–69.

249. Watt C, Abuya T, Warren C, Obare F, Kanya L, Bellows B. Can Reproductive Health Voucher Programs Improve Quality of Postnatal Care? A Quasi-Experimental Evaluation of Kenya’s Safe Motherhood Voucher Scheme. PLOS ONE. 2015;10(4).

250. Wilcox E, Chimedza I, Mabhele S, Romao P, Spiegel J, Zungu M, et al. Empowering Health Workers to Protect their Own Health: A Study of Enabling Factors and Barriers to Implementing HealthWISE in Mozambique, South Africa, and Zimbabwe. INTERNATIONAL JOURNAL OF ENVIRONMENTAL RESEARCH AND PUBLIC HEALTH. 2020;17(12).

251. Woldesenbet S, Kalou M, Mhlongo D, Kufa T, Makhanya M, Adelekan A, et al. An overview of the quality assurance programme for HIV rapid testing in South Africa: Outcome of a 2-year phased implementation of quality assurance program. PLOS ONE. 2019;14(9).

252. Workneh G, Scherzer L, Kirk B, Draper HR, Anabwani G, Wanless RS, et al. Evaluation of the effectiveness of an outreach clinical mentoring programme in support of paediatric HIV care scale-up in Botswana. AIDS care. 2013;25(1):11–9.

253. Wurdeman T, Staffa S, Barash D, Buberwa L, Eliakimu E, Maina E, et al. Surgical Safety Checklist Use and Post-Caesarean Sepsis in the Lake Zone of Tanzania: Results from Safe Surgery 2020. WORLD JOURNAL OF SURGERY. 2022;46(2):303–9.

254. Youngleson MS, Nkurunziza P, Jennings K, Arendse J, Mate KS, Barker P. Improving a mother to child HIV transmission programme through health system redesign: quality improvement, protocol adjustment and resource addition. PloS one. 2010;5(11):e13891.

255. Zeng W, Rwiyereka AK, Amico PR, Avila-Figueroa C, Shepard DS. Efficiency of HIV/AIDS health centers and effect of community-based health insurance and performance-based financing on HIV/AIDS service delivery in Rwanda. The American journal of tropical medicine and hygiene. 2014;90(4):740.
