# Supplementary material for: Trauma-informed care (TIC) in low- and middle-income countries: A scoping review of organisational implementation efforts
Source: Glob Ment Health (Camb). 2025 Dec 10;12:e148. doi: 10.1017/gmh.2025.10111 (PMC12720385; doi:10.1017/gmh.2025.10111)
Supplement: Maiorano et al. supplementary material [file S2054425125101118sup001.zip › Supplementary_File_5._Extraction_template.docx]

S5 Appendix. Extraction template

**Article Information**

Author (Year)

Journal

**Context**

Country(s) of implementation

Other countries involved

If another country was involved, what role did they have (check all that apply)

Funding

Intervention design

Intervention implementation

Intervention evaluation

Intervention dissemination

Other; If other, please specify:

Setting (check all that apply)

Medical (e.g., primary care, hospital)

Mental health (e.g., therapy service)

Educational (e.g., school, university)

Government (e.g., ministry)

Other; If other, please specify:

**QI Aim**

SAMHSA Principle (check all that apply)

Safety

Trustworthiness and transparency

Peer support

Collaboration and mutuality

Empowerment, voice, and choice

Cultural, historical, and gender issues

Cultural, historical, gender issues, please specify:

Gender

Race/ethnicity

Mental health

LGBTQIA+

HIV+

Other; If other, please specify:
