# Supplementary material for: Trauma-informed care (TIC) in low- and middle-income countries: A scoping review of organisational implementation efforts
Source: Glob Ment Health (Camb). 2025 Dec 10;12:e148. doi: 10.1017/gmh.2025.10111 (PMC12720385; doi:10.1017/gmh.2025.10111)
Supplement: Maiorano et al. supplementary material [file S2054425125101118sup001.zip › Supplementary_File_7._Results_Tables.docx]

*Countries of Interventions*

| **Region** | **Country** | **Number of studies (%)** |
| --- | --- | --- |
| East Asia and Pacific |  | 7 (3.03) |
|  | China | 1 (0.43) |
|  | Indonesia | 1 (0.43) |
|  | Papua New Guinea | 1 (0.43) |
|  | Philippines | 3 (1.3) |
|  | Viet Nam | 1 (0.43) |
| Latin America & the Caribbean |  | 25 (10.82) |
|  | Argentina | 1 (0.43) |
|  | Brazil | 8 (3.46) |
|  | Colombia | 2 (0.87) |
|  | Guatemala | 4 (1.73) |
|  | Haiti | 3 (1.3) |
|  | Nicaragua | 5 (2.16) |
|  | Peru | 2 (0.87) |
| Middle East and North Africa |  | 8 (3.46) |
|  | Islamic Republic of Iran | 4 (1.73) |
|  | Jordan | 1 (0.43) |
|  | Lebanon | 2 (0.87) |
|  | Yemen | 1 (0.43) |
| South Asia |  | 44 (19.5) |
|  | Afghanistan | 1 (0.43) |
|  | Bangladesh | 3 (1.3) |
|  | India | 29 (12.55) |
|  | Nepal | 2 (0.87) |
|  | Pakistan | 7 (3.03) |
|  | Sri Lanka | 2 (0.87) |
| Sub-Saharan Africa |  | 147 (63.64) |
|  | Botswana | 3 (1.3) |
|  | Democratic Republic of Congo | 2 (0.87) |
|  | Ethiopia | 6 (2.6) |
|  | Ghana | 12 (5.19) |
|  | Kenya | 20 (8.66) |
|  | Malawi | 16 (6.93) |
|  | Mozambique | 7 (3.03) |
|  | Namibia | 1 (0.43) |
|  | Nigeria | 7 (3.03) |
|  | Rwanda | 4 (1.73) |
|  | Senegal | 3 (1.3) |
|  | Sierra Leone | 3 (1.3) |
|  | South Africa | 21 (9.09) |
|  | Tanzania | 20 (8.66) |
|  | Togo | 1 (0.43) |
|  | Uganda | 10 (4.33) |
|  | Zambia | 11 (4.76) |

*Note: Regions are based on the World Bank’s (2025) classifications.*

*Funding Countries*

| **Funder Country** | **Number of studies (%)** |
| --- | --- |
| Australia | 2(0.66) |
| Belgium | 3(0.99) |
| Brazil | 1(0.33) |
| Canada | 11(3.62) |
| Denmark | 2(0.66) |
| Finland | 2(0.66) |
| France | 2(0.66) |
| Germany | 4(1.32) |
| India | 1(0.33) |
| Ireland | 1(0.33) |
| Italy | 2(0.66) |
| Norway | 2(0.66) |
| South Africa | 1(0.33) |
| Spain | 1(0.33) |
| Sweden | 11(3.62) |
| Switzerland | 4(1.32) |
| The Netherlands | 7(2.30) |
| United Kingdom | 27(8.88) |
| United States | 179(58.88) |
| Unspecified | 41(13.49) |

*Funding Organisations*

| **Funder Organisation Type** | **Number of studies (%)** |
| --- | --- |
| Academic institution | 28(9.21) |
| Business/corporation | 3(0.99) |
| Medical institution | 2(0.66) |
| Multi-country governmental organisation | 27(8.88) |
| National governmental organisation/department/public body | 128(42.11) |
| Nongovernmental organisation/non-profit/not-for-profit/charity | 24(7.89) |
| Private foundation | 70(23.03) |
| Professional body | 7(2.30) |
| Research centre/institute | 3(0.99) |
| Other | 12(3.95) |

*Collaborating Countries*

| **Collaborator Country** | **Number of studies (%)** |
| --- | --- |
| Albania | 1(0.17) |
| Australia | 13(2.21) |
| Belgium | 10(1.70) |
| Brazil | 3(0.51) |
| Canada | 15(2.55) |
| Costa Rica | 2(0.34) |
| El Salvador | 1(0.17) |
| France | 6(1.02) |
| Germany | 10(1.70) |
| Ghana | 1(0.17) |
| India | 1(0.17) |
| Indonesia | 1(0.17) |
| Ireland | 3(0.51) |
| Italy | 6(1.02) |
| Japan | 1(0.17) |
| Kenya | 3(0.51) |
| Lao People's Democratic Republic | 1(0.17) |
| Malawi | 1(0.17) |
| Mexico | 1(0.17) |
| Nigeria | 1(0.17) |
| Norway | 11(1.87) |
| Pakistan | 1(0.17) |
| Poland | 1(0.17) |
| South Africa | 5(0.85) |
| Spain | 4(0.68) |
| Sweden | 23(3.91) |
| Switzerland | 15(2.55) |
| Tanzania | 1(0.17) |
| The Netherlands | 28(4.76) |
| The Philippines | 1(0.17) |
| Uganda | 4(0.68) |
| United Kingdom | 59(10.03) |
| United States | 327(55.61) |
| Unspecified | 23(3.91) |
| Uruguay | 1(0.17) |
| Viet Nam | 1(0.17) |
| Zambia | 2(0.34) |

*Collaborating Organisations*

| **Collaborator Organisation Type** | **Number of studies (%)** |
| --- | --- |
| Academic institution | 299(50.85) |
| Business/corporation | 26(4.42) |
| Medical institution | 45(7.65) |
| Multi-country governmental organisation | 28(4.76) |
| National governmental organisation/department/public body | 43(7.31) |
| Nongovernmental organisation/non-profit/not-for-profit/charity | 99(16.84) |
| Private foundation | 3(0.51) |
| Professional body | 5(0.85) |
| Research centre/institute | 17(2.89) |
| Other | 23(3.91) |

Table 9

*Types of Implementing Organisations*

| **Type of organisation** | **Number of studies (%)** |
| --- | --- |
| Medical (e.g., hospital, clinic) | 231(90.6) |
| Mental health (e.g., therapeutic organisation) | 4(1.60) |
| Educational (e.g., school) | 4(1.60) |
| Governmental (e.g., ministry) | 2 (0.8) |
| Other | 9 (3.5) |
| Multiple | 5(2.0) |
